# Effect of Strain Variations on Lassa Virus Z Protein-Mediated Human RIG-I Inhibition

**DOI:** 10.3390/v12090907

**Published:** 2020-08-19

**Authors:** Qinfeng Huang, Xiaoying Liu, Morgan Brisse, Hinh Ly, Yuying Liang

**Affiliations:** Department of Veterinary and Biomedical Sciences, College of Veterinary Medicine, University of Minnesota, St Paul, MN 55108, USA; huangq@umn.edu (Q.H.); liux2725@umn.edu (X.L.); briss049@umn.edu (M.B.); hly@umn.edu (H.L.)

**Keywords:** Lassa virus, LCMV, arenavirus, sequence variations, Z protein, RIG-I, innate immunity, immune evasion

## Abstract

Mammarenaviruses include several known human pathogens, such as the prototypic lymphocytic choriomeningitis virus (LCMV) that can cause neurological diseases and Lassa virus (LASV) that causes endemic hemorrhagic fever infection. LASV-infected patients show diverse clinical manifestations ranging from asymptomatic infection to hemorrhage, multi-organ failures and death, the mechanisms of which have not been well characterized. We have previously shown that the matrix protein Z of pathogenic arenaviruses, including LASV and LCMV, can strongly inhibit the ability of the innate immune protein RIG-I to suppress type I interferon (IFN-I) expression, which serves as a mechanism of viral immune evasion and virulence. Here, we show that Z proteins of diverse LASV isolates derived from rodents and humans have a high degree of sequence variations at their N- and C-terminal regions and produce variable degrees of inhibition of human RIG-I (hRIG-I) function in an established IFN-β promoter-driven luciferase (LUC) reporter assay. Additionally, we show that Z proteins of four known LCMV strains can also inhibit hRIG-I at variable degrees of efficiency. Collectively, our results confirm that Z proteins of pathogenic LASV and LCMV can inhibit hRIG-I and suggest that strain variations of the Z proteins can influence their efficiency to suppress host innate immunity that might contribute to viral virulence and disease heterogeneity.

## 1. Introduction

Lassa fever is an acute viral illness that is endemic in several western African nations, where it is estimated to infect about 300,000 to 500,000 people annually and to cause 5000 deaths yearly [1]. Currently there are no FDA-approved vaccines and limited treatment options for Lassa fever. The disease is caused by Lassa virus (LASV), a zoonotic RNA virus in the family of *Arenaviridae* with a natural reservoir in the local rodents *Mastomys natalensis, Mastomys erythroleucus*, and African wood mouse *Hylomyscus pamfi* [2,3,4]. Most human infections are asymptomatic. However, about 20% of the cases develop mild to severe symptoms that may lead to hemorrhage, multi-organ failure, and death [5]. The general case-fatality rate is relatively low at approximately 1%, but it can be as high as 15% among hospitalized patients and ~50% in occasional epidemics [6,7,8]. The reasons for this diverse disease heterogeneity are unknown but can be attributed to several factors, such as co-infection with other pathogens, host’s pre-existing immunity, the amount of viral inoculum, and the relatively high genetic diversity among the LASV isolates. 

LASV isolates found in different geographic and host origins are highly diverse in sequences and are phylogenetically classified into four major lineages or clusters (I–IV) with 25% and 32% sequence variations in the viral genomic small (S) and large (L) segments, respectively [9,10]. The L and S genomic segments encode four known viral gene products in opposite orientations [11]. The L segment encodes the large polymerase protein L of ~200 kDa in size and a small matrix protein Z of ~15 KDa. The S segment encodes the nucleoprotein NP (~65 kDa) and the glycoprotein precursor complex (GPC) (~75 kDa). All four of these viral proteins have been shown to be absolutely essential for viral replication and disease pathogenesis in vitro and in vivo (reviewed in [12]). The viral GPC complex mediates viral entry through binding to host cellular receptor and mixing of the viral lipid membrane with that of the cellular endosome in order to release the viral genomic materials into the cytoplasm for viral genomic transcription and replication by the L polymerase and the NP protein (reviewed in [13]). In addition, the viral matrix protein Z has been shown to negatively regulate viral RNA synthesis in order to orchestrate virion assembly and budding at the infected cell surface (reviewed in [14]).

Besides mediating viral genomic RNA encapsidation into the viral ribonucleoprotein (RNP) complex as well as viral RNA transcription and replication, NP has also been shown by us and others to participate in the suppression of IFN-I through its exoribonuclease (RNase) activity to degrade double-stranded immunostimulatory RNA (dsRNA) in order to allow the virus to evade the host immune sensing [15,16], reviewed in [17]). We have also shown that the Z protein of pathogenic arenaviruses including LASV can inhibit IFN-I production by directly binding to and inhibiting the innate immune proteins RIG-I and MDA5, collectively known as the RIG-I-like receptors (RLRs), and in doing so, it acts as a potential viral virulent factor [18]. 

Various animal models have been developed to characterize the degrees of viral virulence and disease pathology mediated by LASV infection (reviewed in [19]). Accumulating evidence has suggested that different LASV isolates can mediate differential degrees of virulence and disease pathogenesis in animal models. The LASV strains isolated from Liberian patients have been shown to cause different degrees of virulence in inbred guinea pigs, although viral virulence in guinea pigs is poorly correlated with the clinical outcomes in humans [20]. A recent study showed that different LASV clinical isolates derived from a patient who suffered from a lethal infection and one from a non-lethal outcome exhibited differential degrees of virulence in the Stat1-/- mouse model that appeared to be consistent with the clinical outcomes observed in the human patients [21]. Additionally, Safronetz and colleagues showed that LASV Soromba-R strain isolated from rodents in Mali was less pathogenic in non-human primates (NHPs) than the human LASV isolates Josiah and Z-132 [22]. We hypothesize that the wide degree of sequence diversity among the LASV isolates may partly account for the differential disease manifestations and pathogenicity. How Lassa viral strain or isolate variations affect viral replication, virulence, immune responses, and pathogenesis have not been well characterized. 

In the current study, we aligned the sequences of the Z protein of different LASV strains and isolates available in GenBank, which showed significant sequence variations at their N- and C- terminal regions. Phylogenetic analysis of the amino acid sequences of these Z proteins shows that they form the same four major clusters as have previously been described for the LASV NP and whole viral genomic nucleotide sequences [9,10]. We chemically synthesized and expressed 20 Z proteins of LASV isolates from human patients and rodent reservoirs, and analyzed their functional activities to inhibit human RIG-I (hRIG-I) by using an established IFN-β promoter-driven luciferase (LUC) reporter assay [18]. Our data show that all tested LASV Z proteins can inhibit hRIG-I, which is consistent with our previous report [18], and that those from human viral isolates are on average slightly stronger inhibitors of hRIG-I than those of the rodent origin. In addition, we showed that Z proteins of four LCMV strains inhibit hRIG-I at different efficiencies. Collectively, our results confirm that Z proteins of LASV and LCMV can strongly inhibit hRIG-I and suggest that strain variations of the Z protein can potentially impact virus-mediated host immune modulation, viral virulence, and disease pathogenesis. 

## 2. Materials and Methods

### 2.1. Sequence Analysis

The full-length amino acid sequences of Z proteins from diverse LASV isolates and LCMV strains were obtained from the GenBank database and were analyzed via multiple protein alignment and phylogenetic tree using the MacVector Software (MacVector, Inc., Apex, NC, USA). 

### 2.2. Cells and Plasmids

Human kidney epithelial 293T cells were grown in Dulbecco’s modified Eagle’s medium (DMEM) supplemented with 10% fetal bovine serum (FBS) and 50 μg/mL penicillin–streptomycin antibiotics. 

Mammalian expression plasmids (pCAGGS) that contain the C-terminally hemagglutinin (HA)-tagged Pichinde virus (PICV), LASV (Josiah), and LCMV (Armstrong) Z proteins were described in our previous publications [18,23]. Expression plasmids for other C-terminally HA-tagged LASV and LCMV Z proteins were similarly constructed using chemically synthesized genes by Genewiz (South Plainfield, NJ, USA) based on the primary sequence obtained from the GenBank database. The list of all Z expression plasmids used in this study is shown in Table 1. The mammalian expression plasmid pEF-Flag-RIG-I-N encoding the N-terminal activation domain of the hRIG-I protein that is fused to a C-terminal FLAG tag was a kind gift of Takashi Fujita (Kyoto University, Japan).

### 2.3. RIG-I-Induced IFNβ-Promoter-Dependent LUC Reporter Assay

293T cells seeded into 12-well plates and grown overnight in the CO_2_ tissue-culture incubator were transfected with either an empty vector or individual Z expression vector at different concentrations (125 ng, 250 ng, 500 ng, or 1000 ng), together with 100 ng of pEF-Flag-RIG-I-N (expressing the N-terminal activation domain of the hRIG-I), 100 ng of IFNβ-LUC vector, which expresses the firefly luciferase (*Fluc*) reporter gene from the DNA regulatory element of the IFN-β promoter, and 50 ng of a β-galactosidase (*β-Gal*)-expressing plasmid for the purpose of normalization for plasmid DNA transfection and RNA polymerase II transcription efficiency. Transfected cell lysates were prepared at 24 h post-transfection. Fluc reporter gene expressions were normalized to the β-Gal gene-expression values as previously described [15]. Each plasmid transfection was conducted in triplicate and repeated in at least two independent experiments. Statistical significance between two groups was determined using the two-tail unpaired Student’s t-test of GraphPad Prism 6 software.

### 2.4. Western Blot

Cell lysates were separated by 15% sodium dodecyl sulfate (SDS)-polyacrylamide gel and transferred onto nitrocellulose membranes. After blocking in 5% nonfat powdered milk in Tris-HCl buffer (TBS), the membrane was incubated with anti-HA and anti-actin monoclonal antibodies (Sigma, St. Louis, MO, USA), washed three times with TBS (50 mM Tris-HCl, pH 7.4), and probed with HRP-conjugated goat anti-mouse antibody (R & D Systems). After washing with TBS buffer with 0.05% Tween 20, the membrane was analyzed by myECL (Thermo Scientific, Waltham, MA, USA) using the enhanced chemiluminescence (ECL) reaction in the Western Chemiluminescent HRP substrate (Millipore Sigma, Burlington, MA, USA).

## 3. Results

### 3.1. Sequence Alignment of Z Proteins from Different LASV Strains and Isolates

**Figure 1:**
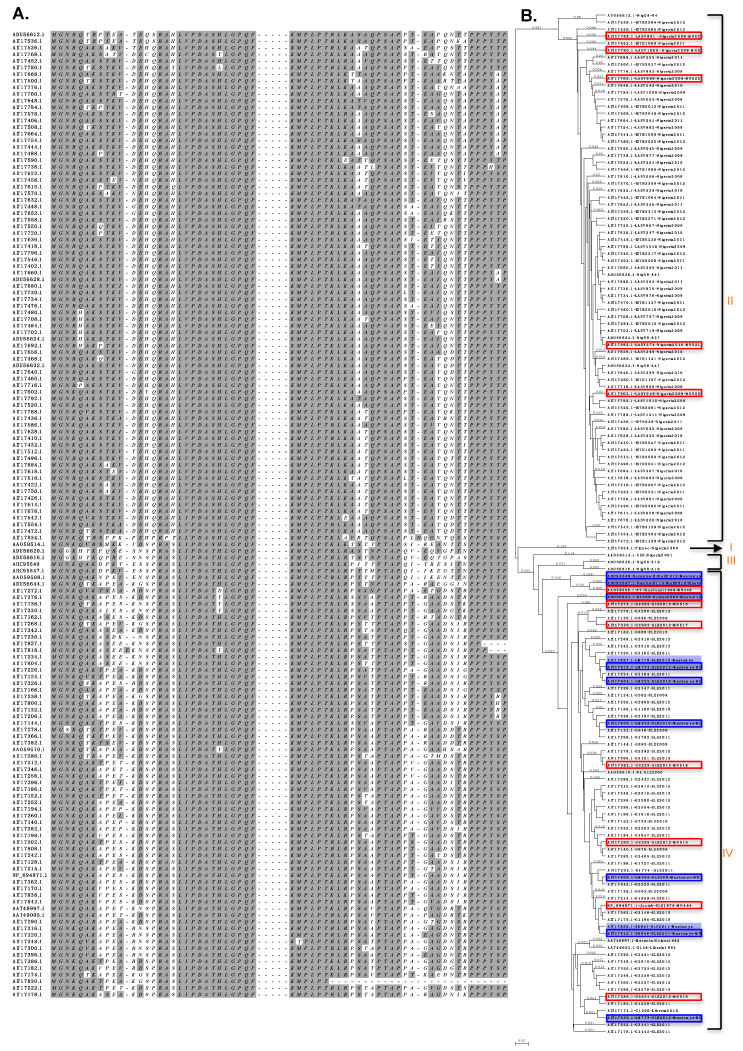
Sequence alignment and phylogenetic analysis of LASV Z proteins. (**A**) The Z protein amino acid sequences obtained from the GenBank database were aligned, with the N-terminal domain (NTD) and the C-terminal domain (CTD) sequences shown. Sequences of the conserved central RING domain are represented as short dash lines. (**B**) The phylogenetic tree based on the amino acid sequences of Z proteins. LASV isolates from rodents (*Mastomys natalensis*) are shown in blue shade. Z proteins analyzed in this study are outlined, those from human viral isolates shown in red boxes and those from rodent viral isolates in blue boxes.

We obtained LASV Z protein sequences from the GenBank database, including those of the Josiah [24], AV, NL, and CSF [25], Macenta and Z148 isolates (deposited by the the Viral Sequencing Group at Lawrence Livermore, 2004), clinical isolates in Nigeria [26], rodent reservoir samples in sub- Saharan Mali [27], and ~200 sequences from clinical and rodent reservoir samples deposited by the Viral Hemorrhagic Fever Consortium [10]. After removing all identical sequences, ~100 unique LASV Z protein sequences were aligned (Figure 1A), which showed as high as 33% sequence variations. Sequence variations occur mostly in the flexible N- and C-terminal domains (NTD and CTD) of Z proteins, while the central RING domain is highly conserved. Phylogenetic analysis (Figure 1B) showed that these Z proteins clustered into the same four major clusters as previously reported based on the LASV NP nucleotide [9] and complete viral genomic sequences [10]. Cluster I included the LASV Pinneo isolate that was originally isolated from an American patient in Nigeria. Clusters II and III consisted of LASV clinical isolates found in Nigeria. Cluster IV included clinical and rodent isolates found in other regions West of Nigeria (Sierra Leone, Guinea, Liberia, Côte d’Ivoire, Mali). Also included in cluster IV were 11 sequences from rodent isolates found in Sierra Leone and Mali (Figure 1B, shaded in blue), which clustered together with human viral isolates and did not form species-specific clusters, consistent with previous finding based on LASV nucleotide sequences [10]. 

### 3.2. Establishment of a Convenient and Quantitative Assay to Evaluate RIG-I Inhibition by Z Proteins

**Figure 2:**
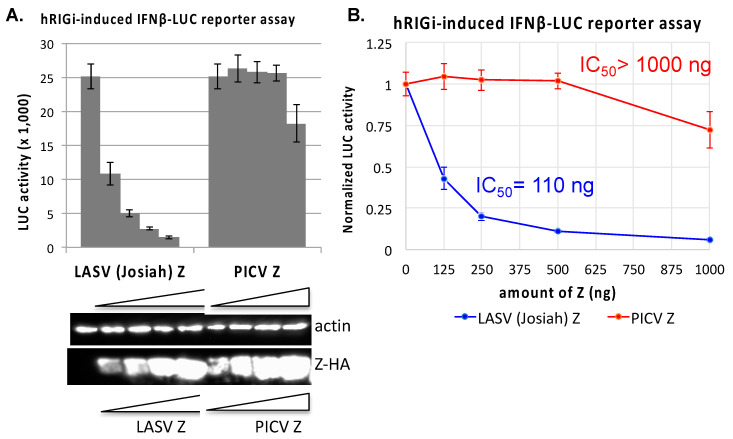
Comparative hRIG-I inhibitory analysis of LASV (Josiah) and PICV Z proteins. The effects of either LASV (Josiah) or PICV Z protein (in a dose-dependent manner) to inhibit the human RIG-I-N-induced IFNβ promoter-driven LUC reporter gene expressions were measured. 293T cells were transfected with a combination of plasmids (pEF-RIG-I-N, IFNβ-LUC reporter plasmid, and a β-Gal expression plasmid) together with either an empty vector or an increasing concentration of the Z expression plasmid. LUC reporter activity was measured at 24 h (**A**) and normalized by that in empty vector control (**B**). The expression of Z proteins in the cells was detected by a Western blot analysis using an anti-HA antibody. IC_50_ value was determined by the amount of the Z plasmid required to reduce the LUC reporter activity by 50%. Each plasmid transfection experiment was conducted in triplicate and repeated in at least two independent experiments.

RLRs such as RIG-I and MDA5 are the major sensors of intracellular RNA virus infection to activate the downstream signaling pathway in order to induce the expression of IFN-I and other proinflammatory cytokines, which together mediate an antiviral response [13]. As such RLRs and their signaling pathways are the frequent targets of viral immune evasion. We have previously shown that Z proteins of pathogenic arenaviruses (e.g., LASV) but not of non-pathogenic arenaviruses (e.g., PICV) can bind to RIG-I and inhibit RLR activation [18]. In order to quantitatively evaluate the ability of the Z protein to inhibit RIG-I function, we used an established human RIG-I-induced IFNβ-LUC reporter assay [28]. Briefly, 293T cells were transfected with a plasmid expressing the N terminal domain of the human RIG-I (hRIG-I-N), which is a constitutively activated form of hRIG-I, and a plasmid that expresses the luciferase (LUC) reporter gene under the regulatory DNA element of the IFNβ promoter (IFNβ-LUC), along with different amounts of the Z protein-expression plasmid. As shown in Figure 2A, under these experimental conditions, LASV (Josiah) Z protein strongly inhibited LUC expression in a dose-dependent manner, which was in sharp contrast to the negative control PICV Z protein that did not significantly reduce the LUC gene expression even at the highest concentration of PICV Z used. The amount of LASV (Josiah) Z plasmid required to reduce LUC gene expression by 50% (IC50) is 110 ng and that for PICV Z is >1000 ng (Figure 2B), which are consistent with our previous report that LASV but not PICV Z can strongly inhibit human RLRs [18]. As the IC50 value is inversely correlated with the ability of the Z protein to inhibit RIG-I function, it can be used to quantitatively evaluate the ability of Z proteins to mediate hRIG-I inhibition.

### 3.3. Evaluation of the Ability of Z Proteins of Different LASV Isolates to Inhibit hRIG-I Function 

**Figure 3:**
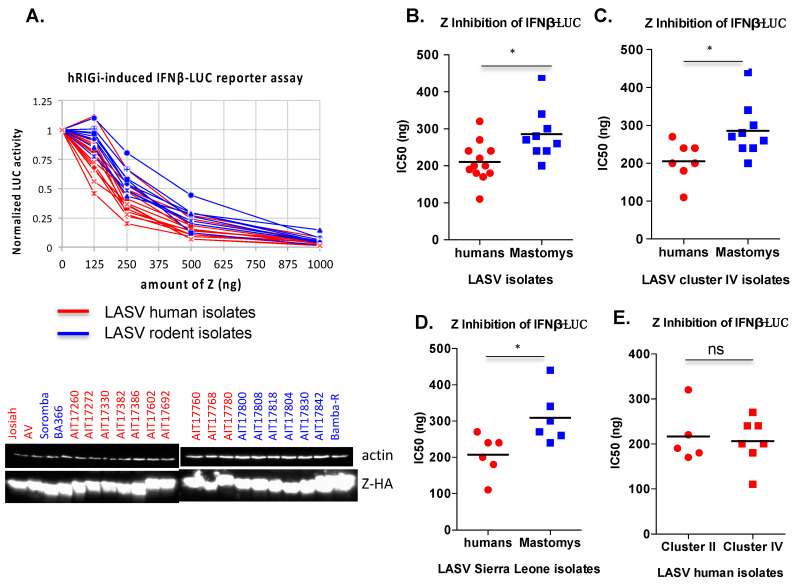
Comparative hRIG-I inhibitory analysis of Z proteins from different LASV isolates. The hRIG-I inhibitory effects by the respective Z proteins (in a dose-dependent manner) were determined as described above in the Figure 2 legend. (**A**) The normalized LUC activity by the empty vector control is shown for each Z protein in different doses. Western blot analysis of the tested LASV Z proteins is shown, with those from human viral isolates in red and those from rodent viral isolates in blue. Notable differences in the size of the Z proteins correspond to differences in their primary amino acid sequences. IC_50_ values were determined by the amount of the Z plasmid required to reduce the LUC reporter activity by 50% and are compared between Z proteins of human and rodent viral isolates (**B**), between Z proteins of human and rodent viral isolates within the same cluster IV (**C**), between Z proteins of human and rodent viral isolates within the same country (Sierra Leone) (**D**), and between Z proteins of human viral isolates between clusters II and IV (**E**). Each plasmid transfection experiment was conducted in triplicate and repeated in at least two independent experiments. Statistical significance between two groups was determined using the two-tail unpaired Student’s *t*-test of GraphPad Prism 6 software. Statistical significance: ns, not significant; *, *p* < 0.05.

As significant sequence variations exist among LASV Z proteins (Figure 1), we asked whether they can impact the ability of LASV Z proteins to inhibit hRIG-I function. To do this, we selected 20 LASV Z proteins that broadly represent the range of sequence variations, chemically synthesized the genes, and cloned them into the pCAGGS mammalian expression plasmid, each with a C-terminal HA tag. These include all nine aforementioned LASV isolates from rodents that belong to cluster IV (Figure 1B, blue box) and 11 randomly selected human LASV isolates from clusters II and IV (Figure 1B, red box). These Z proteins expressed at similar levels in transfected 293T cells as evidenced by Western blotting results using the anti-HA antibody (Figure 3A). 

The ability of each of these Z proteins to inhibit hRIG-I function was examined in the established IFN-β promoter-LUC reporter assay as described in Figure 2. The LASV (Josiah) Z protein was included in each set of the experiments as a reference and a positive control. As shown in Figure 3A, all 20 LASV Z proteins tested could inhibit hRIG-I function in a dose-dependent manner. IC50 was calculated for each of the LASV Z proteins tested. The strongest inhibition is by LASV Z (accession ID AIT17272.1) from a human isolate in Sierra Leone, while the lowest inhibition is by LASV Z (accession ID AIT17808.1) from a *Mastomys* isolate in Sierra Leone (Figure 3A). The IC50 values of Z proteins from rodent (*Mastomys*) LASV isolates were on the average higher than those from human LASV isolates (Figure 3B). As the rodent LASV isolates exclusively belong to cluster IV, while the human LASV isolates belong to clusters II and IV, we decided to compare Z proteins from cluster IV only in order to exclude the potential confounding effect of cluster-specific LASV Z sequence variations. The IC50 values of LASV Z proteins from rodents (*Mastomys*) viral isolates were still higher than those from human LASV isolates of the same cluster IV (Figure 3C). As cluster IV includes isolates from several countries west of Nigeria, we compared only those Z proteins from LASV isolates in Sierra Leone and similarly found higher IC50 values associated with Z proteins of rodent LASV isolates than those of human LASV isolates (Figure 3D). However, when comparing human LASV isolates between clusters II and IV, we did not detect a significant difference in IC50 values (Figure 3E). These results suggest that Z proteins of human LASV isolates are on average stronger inhibitors of hRIG-I than those of LASV isolates found in the natural rodent reservoir (*Mastomys*). 

### 3.4. Evaluation of the Ability of Z Proteins of Different LCMV Strains to Inhibit hRIG-I Function 

**Figure 4:**
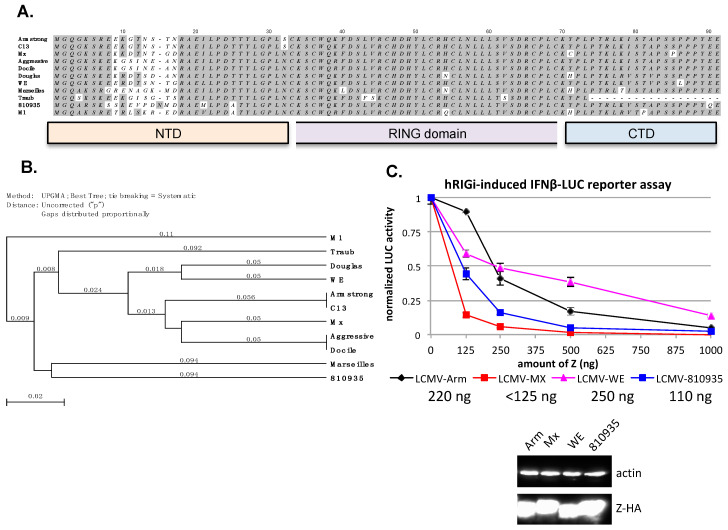
Comparative hRIG-I inhibitory analysis of Z proteins from different LCMV strains. (**A**) The amino acid sequences of Z proteins from different LCMV strains, Armstrong (P18541.3), cl13 (ABC96003), Mx (CAA10342), Aggressive (ACA61299), Docile (ACA61303), Douglas (ACV72588), WE, Marseille #12 (ABB88930), Traub (P19325, partial sequence), 810935(ACV72580), and M1 (BAJ52729) were aligned using the MacVector software, with the N-terminal domain (NTD), the central RING domain, and the C-terminal domain (CTD) shown at the bottom. (**B**) The phylogenetic tree based on the amino acid sequences of Z proteins. (**C**) Comparative hRIG-I inhibitory effects by the respective Z proteins (in a dose-dependent manner) of four different LCMV strains (Armstrong, WE, Mx, and 810935] were determined as described in the legend of Figure 2. Notable differences in the size of the Z proteins correspond to differences in their primary amino acid sequences. The IC_50_ values for each of the LCMV Z proteins tested are shown in the figure legend. Western blot analysis of LCMV Z proteins is shown.

As shown from the above studies, strain variations in the LASV Z protein can modulate its function to inhibit hRIG-I. We wish to extend these findings to another arenavirus, LCMV, with known genetic diversity [29,30]. With a natural reservoir in the house mouse *Mus musculus*, LCMV has a global distribution and causes zoonotic infections that are generally non-symptomatic or mild, but in some cases, result in neurological disorders, spontaneous abortion, congenital defects, and transplant-associated death [31]. Many LCMV strains have been isolated from human patients and rodents, with extensive genetic and phenotypic differences [30]. The Armstrong (Arm) strain was first isolated from an infected patient and has been passaged and used in many laboratories to infect mice for use as an acute viral infection model [32]. The Clone 13 (C13) strain was derived from the Armstrong strain but causes persistent infection in mice [33]. The Traub strain was isolated from a persistently infected mouse in 1935 [34,35]. The WE strain was isolated from an infected patient [36]. The MaTu-Mx (Mx) strain was a new LCMV strain isolated from the MaTu cell line that was presumably derived from a human mammary tumor [37]. The M1 strain was isolated from mice in Japan [38]. The LCMV strain 810935 was derived from human cerebral spinal fluid (CSF) [39]. The Douglas strain was isolated from a human patient [39]. Both the Docile and Aggressive strains of LCMV were derivatives of the parental UBC strain, with Docile causing a persistent infection and Aggressive causing an acute infection in mice [40]. Alignment of these LCMV Z proteins reveals significant sequence variations in the N-terminal domain (NTD) (Figure 4A), and a phylogenetic tree for them was generated (Figure 4B). We previously analyzed the Z protein of LCMV (Arm) for its functional significance to inhibit hRIG-I [18], and decided to compare it to another three LCMV Z proteins. The four LCMV Z proteins, from strains Armstrong (Aim), WE, Mx, and 810935, expressed well in cells after transient transfection. All of them could inhibit hRIG-I in a dose-dependent manner in the established IFNβ promoter-LUC assay (Figure 4C). The inhibitory effect of these LCMV Z proteins is similar between the Arm (IC50 = 220 ng) and WE (IC50 = 250 ng) strains, and stronger for 810935 (IC50 = ~110 ng) and Mx (IC50 < 125 ng) strains (Figure 4C). These results suggest that, similar to LASV Z, strain variations exist among the different LCMV Z proteins and can impact the efficiency of innate immune inhibition.

## 4. Discussion

LASV isolates display high levels of genetic diversity with nucleotide variations up to 32% for the L genomic segments, in sharp contrast to the Ebola viral genome, which is >97% conserved among the sequenced viral isolates and strains [10]. The high levels of genetic diversity among LASV isolates may increase the complexity of viral virulence and disease pathogenesis and present significant challenges in developing effective vaccines and antiviral treatments [30,41]. LASV isolates found in their natural hosts *Mastomys natalensis* cluster together with those isolated from human patients in the same geographical locations, and do not form host-specific clusters [10]. These findings suggest that LASV originated in Nigeria and has spread into western African countries and that the common route of LASV transmissions in humans is mostly rodent-to-human and not human-to-human [10]. Nonsynonymous intra-host protein sequence variations have been found to accumulate in the predicted epitopes of the GPC protein and the variant alleles can significantly reduce GPC binding to the GP1-specific monoclonal antibodies [10], suggesting the strong selective pressure of host antibody response. Another study reveals a major positive selection pressure in the L polymerase protein and/or the NP protein of LASV (and LCMV), indicating both proteins, which make up the core viral polymerase complex, as possible determinant factors of LASV disease severity in humans [42]. However, the roles of viral genetic diversity in LASV virulence and disease pathogenesis have not been fully characterized, especially in a carefully controlled experimental setting.

Despite its small size, the Z protein carries out multiple functions at various steps of the viral life cycle and mediates virus–host interactions through its distinct structural features, which includes an invariable G2 myristoylation site, a highly variable NTD, a conserved central RING domain, and a CTD containing late domain(s) (reviewed in [43]). Z regulates viral RNA synthesis by interacting directly with the L polymerase protein through its central RING domain to block the viral polymerase catalytic activity [23,44,45,46,47]. Z is a key regulator of virion assembly through its G2 site-dependent interaction with viral envelop complex GPC [48], and its RING-domain-dependent interaction with viral ribonucleoprotein (vRNP) components L and NP [46,49,50,51]. Z is the major driving force for virion budding by recruiting proteins of the cellular ESCRT complexes, Tsg101 and Nedd4, through its C-terminal Late domain(s) [23,52,53]. In addition, Z has been found to interact with multiple cellular factors, such as the promyelocytic leukemia protein (PML), ribosomal proteins, and the eukaryotic translation initiation factor 4E (eIF4E), through its central RING domain [54,55,56]. Lastly, Z from pathogenic viruses (e.g., LASV and LCMV) has been shown to interact with hRIG-I through its NTD to inhibit IFN-I induction [18,57]. 

The Z protein has so far been excluded from the evolutionary genetic analysis partly due to its relatively small size (~99 amino acid residues) and its fast-evolving nature [42]. Significant sequence variations occur at both the NTD (residues 8–18) and the CTD (residues 77–93) of LASV Z proteins (Figure 1A), and mostly at the NTD (residues 9–16) of LCMV Z strains (Figure 4A), suggesting that these regions are less likely to be involved in essential activities of the viral life cycle but may potentially affect the function of Z in mediating virus–host interactions with opportunistic hosts such as humans, for example, its interaction with hRIG-I to inhibit induction of IFN-I expression.

In the current study, we examined the potential effects of strain variations within the LASV Z proteins on their ability to inhibit hRIG-I function. We were particularly interested in addressing the question of whether Z proteins of the human LASV isolates show stronger inhibitory effect on hRIG- I than those found in the rodent reservoir. As there were limited numbers of full-length Z protein sequences from LASV rodent isolates available at GenBank and all of them were from cluster IV, we decided to include almost all of them in the analysis. We selected seven Z proteins from human viral isolates in clusters IV, some isolated from the same country in western Africa (Sierra Leone), for a better comparison to rodent viral isolates, as well as five from human viral isolates in cluster II to represent those with the most diversified sequence variations. We showed that all LASV Z proteins tested can inhibit hRIG-I function (Figure 2A), regardless of their host species or geographical locations, confirming our previous findings [18] that inhibition of hRIG-I is a conserved feature among LASV Z proteins. Notably, Z proteins from human viral isolates show a slightly stronger inhibitory effect of hRIG-I on the average than those from rodent viral isolates, even when the comparison is made among viral isolates within the same cluster IV or those in the same country (Sierra Leone) (Figure 2B–D). No significant difference was detected for Z proteins of human viral isolates between cluster II and IV (Figure 2E), despite the fact that the inter-cluster sequence variations are larger than the intra-cluster variations. Although limited by a relatively small sampling size and a limited number of Z sequences available for those viruses isolated from *Mastomys* rodents, our results suggest that sequence variations of LASV Z proteins in a genetically diverse pool of viruses in the rodent hosts can affect their ability to mediate hRIG-I inhibition and that a stronger inhibition of hRIG-I may give the virus an advantage when infecting humans. As the majority of human LASV transmissions are mainly the result of the rodent-to-human and not the human- to- human route [10], viral evolution is thought to be largely determined by events occurring in the rodent reservoirs [42]. However, it remains to be determined whether LASV isolates from rodent reservoirs in other viral clusters (I to III) exhibit any differences in their ability to mediate hRIG-I inhibition, which requires a large-scale sequencing effort to survey LASV pools in rodent reservoirs. Furthermore, little has been known about the mechanisms of innate immunity in *Mastomys* and other natural rodent reservoirs of LASV, and an experimental system is lacking to directly address these important questions. Laboratory mice are of different rodent species and do not persistently shed virus upon LASV infection ([58], reviewed in [19]). As such, we are unable to determine whether LASV Z inhibits innate immunity of their natural rodent hosts in a similar manner or mechanism as seen in humans and whether and how strain variations of the Z protein or other viral proteins may affect LASV interactions within the natural rodent hosts. These are outstanding questions that still need to be addressed to understand LASV evolution and transmission toward the development of effective counter measures to reduce the risk of LASV transmissions in endemic areas. 

It is worth noting that, in addition to the Z protein, LASV contains another IFN-I antagonist—the NP protein—which blocks the IFN-I induction through its conserved 3′–5′ exoribonuclease (RNase) activity [15,16,59]. Recombinant LASV with NP RNase mutants are severely defective in the suppression of IFN-Is in macrophages and dendritic cells [60], demonstrating the importance of LASV NP RNase activity in the IFN-I inhibition. The respective roles of LASV NP RNase and Z in the IFN-I inhibition have not been formally and carefully examined using individual and combined mutations, nor have their roles in host innate suppression and viral virulence been examined in vivo. In contrast to LASV, New World pathogenic arenaviruses, such as Junin and Machupo, induce high level of IFN-Is in the virus-infected cells [61], even though they both have functional NP RNase activity that can strongly inhibit the IFN-I induction in the cell-based gene expression system [59,62], highlighting the complexity of different virus and model systems used in the analysis. In addition, the distinct disease outcomes of LASV infections in humans and in animals suggest a species-specific difference in host innate immunity and its modulation by LASV. Taken together, how IFN-Is are regulated at molecular, cellular, and organismal levels during LASV infections needs to be carefully evaluated, using experimental settings that closely mimic human infections.

## 5. Conclusions

In summary, our study examines the effect of strain variations within LASV Z proteins from different human and rodent isolates to inhibit hRIG-I function that can provide important insights into the role of the Z protein as a virulence factor to mediate host immune evasion upon human LASV infections. Understanding how LASV sequence variations affect the function of viral gene products and host immune responses is critical for understanding disease heterogeneity and for the development of effective vaccines and antivirals with a broad protection against different LASV natural isolates [41].

## Figures and Tables

**Table 1:** List of the Z protein sequences and their expression plasmids used in this study.

Z Plasmid Name	Accession Number	Virus	Isolate/Strain	Host Species	Country Isolated	LASV Cluster
MN444	NP_694871.1	LASV	Josiah	*Homo sapiens*	Sierra Leone	IV
MN446	AAO59508.1	LASV	AV	*Homo sapiens*	Ivory Coast	IV
MN447	AHC95549.1	LASV	Soromba-R	*Mastomys natalensis*	Mali	IV
MN496	ADU56644.1	LASV	BA366	*Mastomys natalensis*	Guinea	IV
MN515	AIT17260.1	LASV	G2295	*Homo sapiens*	Sierra Leone	IV
MN516	AIT17272.1	LASV	G2363	*Homo sapiens*	Sierra Leone	IV
MN517	AIT17330.1	LASV	G2903	*Homo sapiens*	Sierra Leone	IV
MN518	AIT17382.1	LASV	G3229	*Homo sapiens*	Sierra Leone	IV
MN519	AIT17386.1	LASV	G3234	*Homo sapiens*	Sierra Leone	IV
MN520	AIT17602.1	LASV	LASV049	*Homo sapiens*	Nigeria	II
MN521	AIT17692.1	LASV	LASV274	*Homo sapiens*	Nigeria	II
MN522	AIT17760.1	LASV	LASV989	*Homo sapiens*	Nigeria	II
MN523	AIT17768.1	LASV	LASV991	*Homo sapiens*	Nigeria	II
MN524	AIT17780.1	LASV	LASV1000	*Homo sapiens*	Nigeria	II
MN525	AIT17800.1	LASV	LM032	*Mastomys natalensis*	Sierra Leone	IV
MN526	AIT17808.1	LASV	LM395	*Mastomys natalensis*	Sierra Leone	IV
MN527	AIT17818.1	LASV	LM774	*Mastomys natalensis*	Sierra Leone	IV
MN528	AIT17804.1	LASV	LM222	*Mastomys natalensis*	Sierra Leone	IV
MN529	AIT17830.1	LASV	LM779	*Mastomys natalensis*	Sierra Leone	IV
MN530	AIT17842.1	LASV	Z0948	*Mastomys natalensis*	Sierra Leone	IV
MN531	AHC95547.1	LASV	Bamba-R114	*Mastomys natalensis*	Mali	IV
MN445	ABU39910	PICV	Munchique CoAn4763 P2/P18 isolate	*Cavia porcellus*	lab strain	
MN448	P18541.3	LCMV	Armstrong	*Homo sapiens*	lab strain	
MN915	CAA10342	LCMV	MX	MaTu cell line	lab strain	
MN916	AAD03395.1	LCMV	WE	*Homo sapiens*	lab strain	
MN917	ACV72580	LCMV	810935	*Homo sapiens*	USA	

## Abbreviations

LCMV	lymphocytic choriomeningitis virus
LASV	Lassa virus
IFN-I	type I interferon
LUC	luciferase
NP	nucleoprotein
GPC	glycoprotein precursor complex
RNP	ribonucleoprotein
RNase	exoribonuclease
RLRs	RIG-I-like receptors
NHPs	non-human primates
PICV	Pichinde virus
Fluc	firefly luciferase
ECL	enhanced chemiluminescence
NTD	N-terminal domain
CTD	C-terminal domain
CSF	cerebral spinal fluid
